# The Living the Example Social Media Substance Use Prevention Program: A Pilot Evaluation

**DOI:** 10.2196/mental.7839

**Published:** 2017-06-27

**Authors:** William Evans, Elizabeth Andrade, Sandra Goldmeer, Michelle Smith, Jeremy Snider, Gunilla Girardo

**Affiliations:** ^1^ Milken Institute School of Public Health Department of Prevention and Community Health The George Washington University Washington, DC United States; ^2^ Mentor Foundation USA Washington, DC United States; ^3^ School of Public Health University of Washington Seattle, WA United States

**Keywords:** substance use prevention, peer-to-peer education, social media, adolescence

## Abstract

**Background:**

Adolescent substance use rates in rural areas of the United States, such as upstate New York, have risen substantially in recent years, calling for new intervention approaches in response to this trend. The Mentor Foundation USA conducts the Living the Example (LTE) campaign to engage youth in prevention using an experiential approach. As part of LTE, youth create their own prevention messages following a training curriculum in techniques for effective messaging and then share them via social media. This paper reports on a pilot evaluation of the LTE program.

**Objective:**

To conduct a pilot test of LTE in two rural high schools in upstate New York. We hypothesized that positive antidrug brand representations could be promoted using social media strategies to complement the Shattering the Myths (STM) in-person, event-based approach (hypothesis 1, H1), and that youth would respond positively and engage with prevention messages disseminated by their peers. We also hypothesized that exposure to the social media prevention messages would be associated with more positive substance use avoidance attitudes and beliefs, reductions in future use intentions, and decreased substance use at posttest (hypothesis 2, H2).

**Methods:**

We adapted a previously published curriculum created by the authors that focuses on branding, messaging, and social media for prevention. The curriculum consisted of five, one-hour sessions. It was delivered to participating youth in five sequential weeks after school at the two high schools in late October and early November 2016. We designed a pre- and posttest pilot implementation study to evaluate the effects of LTE on student uptake of the intervention and short-term substance use and related outcomes. Working at two high schools in upstate New York, we conducted a pilot feasibility evaluation of LTE with 9th-grade students (ie, freshmen) at these high schools. We administered a 125-item questionnaire online to capture data on media use; attitudes toward social media; next 30-day personal drug use intentions; personal reasons to use drugs; reasons participants believe their peers would use drugs; self-reported exposure to the LTE program; and receptivity to the LTE program, among those reporting exposure. We constructed multivariable logistic regression models to analyze the relationship between program receptivity and outcomes. First, in a cross-sectional logistic regression model, we regressed self-reported LTE message receipt on drug use intent and actions related to LTE messaging. Then, for analysis of participants with matched pre- and posttest responses, we used multilevel generalized estimating equation (GEE) techniques to model changes in behavior from baseline to follow-up.

**Results:**

Youth reported increased intentions to use marijuana (odds ratio [OR] 2.134, *P*=.02) between pre- and posttest. However, youth who reported exposure and receptivity to LTE reported a significant decrease in intentions (OR 0.239, *P*=.008). We observed a similar pattern for sedatives/sleeping pills—an increase in intentions overall (OR 1.886, *P*=.07), but a decrease among youth who reported exposure and receptivity to LTE (OR 0.210, *P*=.02). We saw the same pattern for use of any drug—an increase in reported intentions overall (OR 2.141, *P*=.02), but a decrease among youth who reported exposure and receptivity to LTE (OR 0.111, *P*=.004).

**Conclusions:**

We observed some evidence of significant LTE program effects. Social media may be an effective strategy for peer-to-peer substance use prevention in the future. These findings point both to the potential of LTE and the social media diffusion model and to the need for more research on a larger scale with an expanded youth population in the future.

## Introduction

### Background

Adolescent substance use rates in rural areas of the United States, such as upstate New York, have risen substantially in recent years [1], calling for new intervention approaches in response to this trend. There is growing evidence that substance use, including marijuana and other drug use, has negative health consequences for adolescents, especially when use begins early and when multiple substances are used [2]. Recent studies suggest adolescent marijuana use may be linked to altered longer-term neurodevelopmental trajectories, compromised neural health, impaired frontal lobe function, and psychosocial effects [3-8]. Additionally, early-onset adolescent marijuana use combined with alcohol and other substance use has been linked to numerous cognitive impairments and neural health effects [9,10]. Social norms favoring many forms of substance use are increasing [11,12] and may be associated with medicalization and legalization of marijuana in some states [13,14]. According to the 2016 survey Monitoring the Future—a long-term study of the behaviors, attitudes, and values of American adolescents, college students, and young adults—38% of high school seniors living in states with medical marijuana laws reported past-year use, compared with 33% in states without these laws. Furthermore, perceptions regarding the dangers of marijuana are at the lowest point ever, with only 31% of high school seniors perceiving smoking marijuana regularly as a “great risk.” Increased adolescent substance use due to changing norms and relaxed laws is a substantial public health threat.

The Mentor Foundation USA conducts the Living the Example (LTE) campaign [15], which includes an interactive youth rally event, Shattering the Myths (STM), designed to dispel myths surrounding drug abuse and engage youth in prevention messages using an experiential learning approach. LTE is a branded program that creates new mental associations with the positive attributes of avoiding substance use that may modify adolescent social norms and reduce drug use intentions [16]. The rally is a catalyst for youth to become advocates for prevention; however, the rally was originally conceived to be conducted in person, thus limiting potential reach of prevention messages. In response to the growing popularity and use of social media, we adapted LTE to include a social media component. We trained youth advocates to create LTE-branded prevention messages, disseminate them via social media platforms, and engage peers in their social networks, with the intention of increasing peer interaction around the brand’s core messaging. We conducted a pilot study in Columbia County, New York, to evaluate the efficacy of LTE with the added social media component. We also assessed the utility of this novel approach using social media strategies and branding principles to reach at-risk youth with prevention messages, engage youth in the program’s brand, and monitor exposure to specific social media channels.

### Potential of Social Media for Prevention

New technologies, including the Internet, social media, and mobile phones, offer tremendous potential to expand the reach and effectiveness of public health programs [17,18]. As noted, some prevention programs have used social media as delivery channels, such as Above the Influence with its large Facebook presence and efforts to create a social community of youth sharing narratives related to the avoidance of marijuana [18-20]. However, relatively little has been published demonstrating the effectiveness of social media as substance use prevention channels. The Substance Abuse and Mental Health Services Administration (SAMHSA) has funded a number of statewide media campaigns for prevention, some of which have used social media activities, including Colorado’s SpeakNow! Campaign focused on teen drinking prevention [21]. However, these efforts are in their infancy, and LTE is a novel effort to design and test a systematic intervention for prevention driven by social media.

### Theoretical Basis for Living the Example: Branding and Social Media

Schools are a common context for interventions, given their almost universal access to youth. School-based interventions have an established history with an emerging array of successful interventions documented on SAMHSA’s National Registry of Evidence-based Programs and Practices [20]. Reviews report an average effect size for youth in school substance use prevention programs in the range of Cohen *d*=0.10 to 0.16 [22-27]. However, in a prevention environment in which marijuana use—and potentially other substance use—is normalized, a more comprehensive approach using other channels to deliver prevention messages is needed. Given its near ubiquity, one promising channel is social media.

Previous research provides a basis for adding media to school interventions. In the conceptual framework behind Slater and colleagues’ intervention that combined in-school activities and community-level media, Be Under Your Own Influence, adolescent experience was embedded in school, community, and the larger social world experiences [28]. Other prevention efforts have been conducted in rural communities and school settings, similar to LTE, and have demonstrated effectiveness [29-31].

LTE provides an even broader community reach by offering a strong presence on social media platforms widely used by adolescents. This approach complements and extends the reach of the existing Mentor Foundation USA‘s in-person STM youth rally. Social media provides access to a larger social world that is inaccessible via direct experience [32]. Social media messages echo branded STM rally messages beyond school walls, reinforcing and amplifying antiuse norms [33].

**Figure 1:**
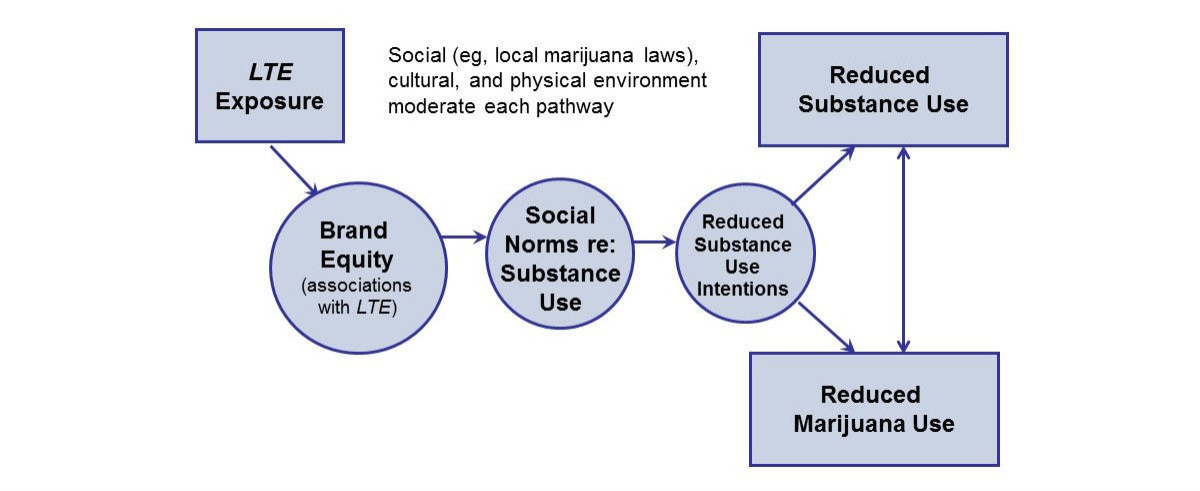
Living the Example (LTE) conceptual model.

Health branding represents an evolution in behavioral theory, building on social cognitive theory (SCT) and the theory of planned behavior (TPB) (see Figure 1) [34,35]. Health branding specifies the modeling component of SCT by proposing a testable process by which the benefits of healthy behaviors may be depicted through positive social role models, such as teens who remain drug-free and thereby achieve social status and respect. It also specifies the attitude component of TPB, namely that a change in attitudes targeted by health messages is mediated by the novel theoretical construct of brand equity (see Figure 1). Health branding extends research on the mediation of health beliefs targeted in behavior change campaigns [36]. Previous research on prevention programs such as Above the Influence demonstrates that higher brand equity is associated with improved antiuse attitudes and norms [18]. This study extends that research.

Using social media strategies and branding principles, we conducted a pilot test of LTE in two rural high schools in upstate New York. We hypothesized that positive antidrug brand representations could be promoted using social media strategies to complement the STM in-person, event-based approach (hypothesis 1, H1), and that youth would respond positively and engage with prevention messages disseminated by their peers. We also hypothesized that exposure to the social media prevention messages would be associated with more positive substance use avoidance attitudes and beliefs, reductions in future use intentions, and decreased substance use at posttest (hypothesis 2, H2).

## Methods

### Intervention

In late September and early October 2016, we conducted a weeklong, in-person STM rally at each school based on the idea of Living the Example (ie, living drug-free as a positive alternative to drug use) at two high schools in Columbia County, New York. Following the weeklong rally, we engaged a group of youth ambassadors (n=12 per high school) in a 5-week, after-school social media and prevention-branding training activity. As part of the training, the ambassadors learned how to develop and disseminate their own prevention messages. They were trained to create social media content and share their drug use avoidance experiences, thus forming positive antidrug social norms with their friends and social networks. The training was based on a previous activity developed by the two lead authors (WE and EA) under National Institutes of Health funding. For 5 weeks after the weeklong STM rally, ambassadors at both high schools disseminated prevention messages through their social networks with the #livingtheexample hashtag to identify posts as representing the LTE program.

### Living the Example Social Media Training

The social media training consisted of five, one-hour sessions. The training was conducted five sequential weeks after school at the two high schools in late October and early November 2016. The curriculum comprised the following:

1. Session 1: What is a Brand? This session described the idea of branding; how it is used to market products, services, and companies; and how it can be applied to social causes and behavior change. The idea of branding substance use prevention was introduced.

2. Session 2: Introduction to Social Media. This session introduced youth to the basics of social media, how it can influence message recipients, and how to begin thinking about creating their own influential messages. The session included a social media message creation exercise.

3.  Session 3: Boosting Online Engagement. This session covered how to connect and build engagement with social networks. It also covered the idea of social media as a conversation, knowing one’s audience, techniques to create engaging posts, and how to communicate about prevention topics with peers.

4. Session 4: Using Your Voice—Introduction to Advocacy. This session focused on how youth can share their opinions about an issue in their community that they would like to change. It examined examples of how advocacy has made a difference in social causes and how to create advocacy messages.

5.  Session 5: Advocacy in Action. This session focused on applying concepts from the preceding sessions to advocate for substance use prevention with peers. It included an exercise in creating a persuasive social media prevention message and post.

Once the training was completed, youth were encouraged to continue creating their own prevention messages and disseminating them to peers through their preferred social media channel for the rest of the fall 2016 semester.

### Evaluation Methods

We designed a pretest-posttest pilot implementation study to evaluate the effects of LTE on student uptake of the intervention and short-term substance use and related outcomes. Working at two high schools in upstate New York, we conducted a pilot feasibility evaluation of LTE with 9th-grade students (ie, freshmen) at these high schools. The rationale for testing the program with freshmen was that they had not yet been enrolled in any previous high school-level prevention programs, including Mentor Foundation USA programs. Due to challenges in collecting posttest data at one high school, the following presentation of data and results focuses on one school for which we successfully completed both pre- and posttesting. We sought to evaluate whether branded prevention messages disseminated via social media increased intervention effects of the adolescent substance use prevention program.

### Measures and Instrument

We developed a questionnaire using validated scales from previous work by the authors [37,38], as well as from other validated scales from both the SAMHSA 2014 Communities that Care survey instrument and the 2012 Monitoring the Future survey [39,40]. The 125-item instrument was programmed into SurveyMonkey software for computer-administered completion during a required freshman English class at both high schools. In addition to demographic information and last grade completed in school, other scales used included the following: Traditional and Digital Media Use (9 items); Attitudes Toward Social Media (18 items); Drug Use Risk Perceptions (12 items); Personal and Perceived Peer Reasons to Use Drugs (6 items); Drug Use Social Norms (18 items); Perceived Peer Drug Use (18 items); Reported Peer Drug Use (14 items); Self-Reported Past 30-Day Drug Use (14 items); Next 30-Day Drug Use Intentions (8 items); Drug Use/Refusal Influences (8 items); and Self-Reported Exposure to the LTE Program and Receptivity to the LTE Program (7 items), which was administered among those reporting exposure.

### Data Collection

We recruited participants from the 9th-grade student bodies at the two high schools and attempted to obtain full participation from all freshmen. Active parental consent had been previously obtained for youth ambassadors to participate in the social media training activity and we sought passive parental consent for all potential freshmen participants in the questionnaire. Youth informed assent was also obtained prior to questionnaire administration. No personal contact or other identifying information was stored with the questionnaire data and a unique identifier was created and used to match pre- and postquestionnaire responses by participant. All study instruments and the protocol were approved by the George Washington University Institutional Review Board and the principals of each high school.

Pretest questionnaires were administered before the intervention launched in late September 2016 and posttest questionnaires were administered in December 2016. Students were asked to log into a password-protected site and complete the pretest and posttest questionnaires online using SurveyMonkey. A total of 129 participants were recruited at the high school included in the pre-post analysis, representing the entire eligible freshman student body at that school. The questionnaire was anonymous and confidential; no student was obligated to complete the questionnaire or penalized for nonparticipation. Students were enrolled in a contest for retail gift card prizes as an incentive upon completion.

### Data Analysis

We conducted all data analysis in Stata release 14 (StataCorp LLC). For each wave of the study, we matched respondents and were able to identify matching records for 80 of the 129 (62.0%) total study participants; 49 students out of 129 (38.0%) completed the posttest only. We compared drug use intent; personal reasons why they, or general reasons why their peers, might use drugs; and agreement with actions related to LTE messaging for baseline and follow-up questionnaires. We also examined respondent exposure to both traditional and new media, social media use attitudes, and how students interacted with LTE social media posts. We created dummy variables that represented students’ likelihood of using a specific or any drug in the next 30 days. We also created a dummy variable to represent whether the respondent self-reported receipt of LTE social media posts.

We then constructed multivariable logistic regression models to analyze the relationship between program receptivity and outcomes. First, in a cross-sectional logistic regression model, we regressed self-reported LTE message receipt on drug use intent and actions related to LTE messaging. Then, for analysis of participants with matched pre- and posttest responses, we used multilevel generalized estimating equation (GEE) techniques to model changes in behavior from baseline to follow-up. We estimated the odds of reductions in drug use intent from baseline to follow-up in those who interacted with LTE compared to those who did not. All models included age and gender as covariates.

**Table 1:** Descriptive statistics of participants (n=80).

Characteristics		n (%)
**Gender**		
	Female	39 (49)
	Male	40 (50)
	Other/transgender	1 (1)
	Total	80 (100)
**Age at baseline (years)**		
	13	9 (11)
	14	64 (80)
	15	6 (8)
	16	1 (1)
	Total	80 (100)

## Results

Table 1 summarizes the sample demographics of freshmen successfully surveyed at follow-up.

**Figure 2:**
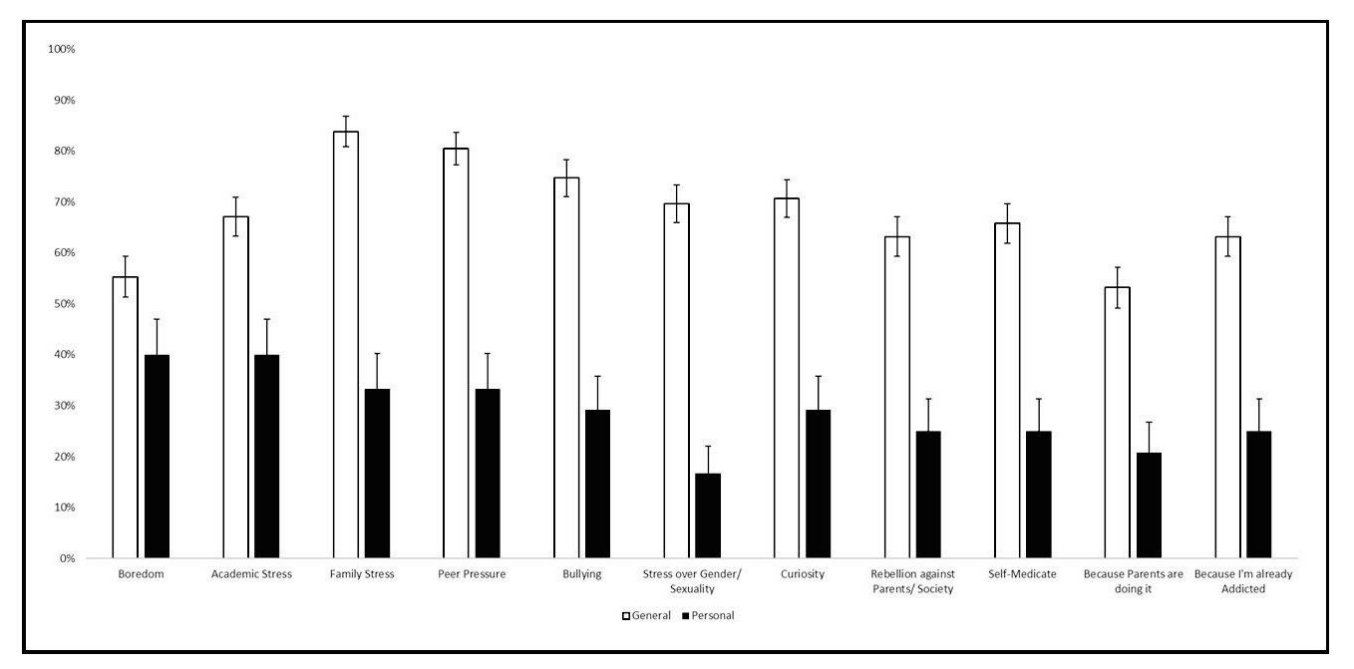
Reasons for peer and personal drug use among matched respondents.

Figure 2 summarizes results on reasons why participants believed their peers used drugs. Nearly all of the categories of reasons scored above 50%, indicating that youth had many reasons why they believed their peers would use drugs. Peer pressure showed up as the most commonly reported reason (36/49, 73%) among participants who only responded to the wave 2 questionnaire. Among those who responded to both waves, family stress was the most common reason (67/80, 84%). The most common overall reason for drug use among all respondents was family stress (105/129, 81.4%).

Figure 2 also summarizes reasons why participants said that they personally used drugs. As personal reports of drug use are generally lower, the results for this scale were lower than perceptions of peer use. Among those who responded to both waves, boredom and academic stress were the most common reasons (32/80, 40%). The same two categories were most common among all respondents (43/129, 33.3%).

**Table 2:** Multivariate regressions comparing self-reported drug use intent at pre- and posttest and Living the Example receptivity (matched participants, n=80).

	Self-reported drug use intent, exponentiated coefficient (*P*)													
	1^a^	2^b^	3^c^	4^d^	5^e^	6^f^	7^g^	8^h^	9^i^	10^j^	11^k^	12^l^	13^m^	14^n^
Any LTE^o^ exposure	0.918 (.94)	0.565 (.61)	2.445 (.18)	0.678 (.74)	1.491 (.61)	0.972 (.96)	1.217 (.86)	2.097 (.45)	2.097 (.45)	2.097 (.45)	2.364 (.38)	2.097 (.45)	2.360 (.24)	2.121 (.19)
Change from baseline to follow-up	1.672 (.35)	1.892 (.06)	1.886 (.07)	2.261 (.08)	1.848 (.17)	2.134 (.02)	1.681 (.15)	1.066 (.90)	1.066 (.90)	1.066 (.90)	0.855 (.76)	1.066 (.90)	0.676 (.45)	2.141 (.02)
Change from baseline to follow-up among those with LTE receptivity	1.820 (.54)	1.495 (.62)	0.210 (.02)	1.409 (.72)	0.432 (.33)	0.239 (.008)	1.809 (.35)	1.000 (>.99)	1.000 (>.99)	1.000 (>.99)	1.000 (>.99)	1.000 (>.99)	0.736 (.73)	0.111 (.004)
Gender^p^	0.663 (.63)	3.027 (.12)	1.343 (.60)	0.845 (.81)	1.197 (.76)	1.698 (.40)	1.279 (.77)	1.108 (.91)	1.108 (.90)	1.108 (.91)	1.699 (.54)	1.108 (.91)	1.691 (.43)	2.006 (.12)
Age	0.467 (.28)	0.560 (.24)	0.614 (.29)	0.428 (.17)	0.508 (.22)	0.834 (.70)	0.462 (.12)	0.782 (.74)	0.782 (.74)	0.782 (.74)	0.581 (.44)	0.782 (.74)	0.740 (.60)	0.673 (.33)

^a^Will smoke cigarettes.

^b^Will use electronic cigarettes (ie, vaping).

^c^Will use sedatives such as sleeping pills.

^d^Will use tranquilizers or antianxiety drugs.

^e^Will use painkillers such as OxyContin or similar.

^f^Will use marijuana.

^g^Will use synthetic marijuana or K2/Spice.

^h^Will use cocaine.

^i^Will use crack.

^j^Will use hallucinogens.

^k^Will use any inhalant for kicks or to get high.

^l^Will use heroin.

^m^Will use any other medicines or substances.

^n^Will use at least one drug.

^o^LTE: Living the Example.

^p^Female is the reference for gender.

Table 2 summarizes results of the GEE models we developed to compare pre- and posttest results and the effect of self-reported exposure and receptivity to LTE social media messages. Youth reported increased intentions to use marijuana (odds ratio [OR] 2.134, *P*=.02) between pre- and posttest, which may be expected given the age range of 14-15 years and concomitant increase in drug use intentions observed in other research for this age group [41,42]. However, among youth who reported exposure and receptivity to LTE, they reported a significant decrease in marijuana use intentions (OR 0.239, *P=*.008). We observed a similar pattern for sedatives/sleeping pills—an increase, although only marginally significant, in intentions overall (OR 1.886, *P=*.07), but a decrease among youth who reported exposure and receptivity to LTE (OR 0.210, *P=*.02). We saw the same pattern for use of any drug—an increase in reported intentions overall (OR 2.141, *P=*.02), but a decrease among youth who reported exposure and receptivity to LTE (OR 0.111, *P*=.004). No other statistically significant results were observed, although a marginally significant increase in e-cigarette (ie, vaping) use was observed among all respondents (OR 1.892, *P*=.06) and a nonsignificant increase was observed among those exposed and receptive to LTE (OR 1.495, *P*=.62).

## Discussion

### Principal Findings

With respect to H1, we found that, overall, youth responded positively and engaged with LTE messages when they were exposed to them by their peers. As shown in Table 2, message receptivity was generally high among those who self-reported exposure to LTE social media. Respondents found LTE to be engaging and convincing, and they generally liked the posts. In terms of immediate response from the target audience, the LTE peer-to-peer approach appears to be a promising way to deliver prevention messages.

We also confirmed H2 in that positive receptivity to LTE messages was associated with some evidence of reduced self-reported drug use intentions, specifically for marijuana and use of sedatives/sleeping pills, and reports of intent to use any drug. As shown in Table 2, the overall sample showed a significant increase in intent to use both marijuana, sedatives/sleeping pills, and any drug, but there was a significant reduction in intent among those who were receptive to LTE messages. While this result could be due to other factors not measured in the study, given the pilot nature of this work and intent to establish preliminary evidence of efficacy and feasibility, these findings suggest that LTE is promising.

Additionally, we identified a number of key reasons why youth believe their peers use drugs and why they personally would use drugs. The most frequently cited reasons why youth believe their peers use drugs and why they themselves would use drugs were academic stress, family stress, and peer pressure. In an overall social environment where marijuana use laws are relaxing, perceived risk and social unacceptability of marijuana use may be decreasing [43]. These changing perceptions and risk factors should be investigated in future studies.

We observed no significant effects for a number of other drugs measured, including cigarettes, e-cigarettes, prescription drugs, inhalants, cocaine, and others. However, the LTE curriculum did not specifically focus on these drugs and in conversations with youth (see sections below), we found that youth did not post about them specifically. Thus, message recipients’ attitudes toward these drugs may not have been affected.

Overall, study findings suggest that peer-to-peer substance use prevention via social media is a promising strategy. Given the low cost and low burden of social media as an intervention channel, schools, communities, and prevention programs can use this approach even in low-resource settings. However, more research is needed on how best to structure such programs. LTE used a model that combined a curriculum, training of peer leaders, and sharing of prevention messages in a social network by those trained peer leaders. The advantages, disadvantages, and alternatives to this model should be explored in future studies.

### Intervention Challenges and Opportunities

In terms of the process of implementing the LTE intervention and the social media training, we observed many positive aspects of the intervention, as well as some challenges. Youth ambassadors who received the social media and peer leadership training liked the experience, were receptive to LTE overall, and reported enjoying the program. Based on our survey data, we have evidence that they participated and did indeed share sufficient social media posts with their peers to generate high LTE awareness in their social networks and produce the observed effects on substance use intentions. Additionally, we also gathered valuable information on how best to use social media platforms. Students indicated that they frequently used Snapchat Geofilters to stay connected to peers. LTE and similar programs may benefit from using this tool, including at events and within social media training/school programming. Furthermore, Instagram and Snapchat now have 24-hour *Story* features that youth encouraged us to use. These features allow more people to see the posts for a longer period of time, thus potentially facilitating diffusion of messages, enhancing reach, and increasing exposure to program messages.

We experienced some challenges with the LTE social media training. One was that our social media examples were often based on Facebook, which is a platform that many youth participants were no longer using. Alternatively, we found that Snapchat and Instagram were the most widely used platforms, with Snapchat typically being used for person-to-person contact (ie, one individual at a time, similar to texting) and Instagram being used for *posts*. Almost none of the participants used Facebook and very few had a Twitter account. Use of Snapchat and Instagram presented challenges for the program, since they were less conducive to detailed posts that tend to be best for prevention advocacy. We recognize that future versions of LTE and programs using similar engagement strategies need to be responsive to rapidly changing social media use patterns among adolescents.

In addition to the social media platform used, youth participants indicated that the intention of the social media message should match the purpose for which they usually used a particular platform. In other words, youth participants primarily used Instagram to demonstrate personal involvement or creativity, and not necessarily to send messages, including prevention messages.

However, participants were more willing to put out *motivational messages* that might deter a peer from using drugs. These messages were intended to be positive and could show “who they were.” The research team faced some challenges communicating and reminding the youth ambassadors to post on social media. We attempted to use direct messaging through Instagram, but some students reacted negatively, indicating a preference for not receiving contact from school or adults through the platform, which they considered as an extension of their “personal space” reserved for socializing with peers.

### Limitations

Finally, it should be noted that this was a pilot study and had the limited objective of demonstrating the potential of social media as a peer-to-peer education tool for prevention. As such, we had a relatively small sample size and, thus, limited statistical power. Therefore, our results should be interpreted with caution. The sample was limited to freshmen; while we achieved a near census of full participation among the freshman class, this did not represent the school as a whole. Additionally, results are not generalizable beyond this population or the individual school setting.

Despite these limitations, LTE did demonstrate a significant program effect. Social media may be an effective strategy for peer-to-peer substance use prevention in the future. Anecdotal information gathered during implementation revealed a number of ways the program and use of social media may be optimized in the future. These findings point both to the potential of LTE and the social media diffusion model and to the need for more research on a larger scale with an expanded youth population in the future.

## Abbreviations

GEE: generalized estimating equation
H1: hypothesis 1
H2: hypothesis 2
LTE: Living the Example
OR: odds ratio
SAMHSA: Substance Abuse and Mental Health Services Administration
SCT: social cognitive theory
STM: Shattering the Myths
TPB: theory of planned behavior

## References

[1] (2017). New York State Department of Health.

[2] Lisdahl KM, Gilbart ER, Wright NE, Shollenbarger S (2013). Dare to delay? The impacts of adolescent alcohol and marijuana use onset on cognition, brain structure, and function. Front Psychiatry.

[3] Jacobus J, Squeglia LM, Sorg SF, Nguyen-Louie TT, Tapert SF (2014). Cortical thickness and neurocognition in adolescent marijuana and alcohol users following 28 days of monitored abstinence. J Stud Alcohol Drugs.

[4] Evans WD, Hastings G, Evans WD, Hastings G (2008). Public health branding: Recognition, promise, and delivery of healthy lifestyles. Public Health Branding: Applying Marketing for Social Change.

[5] Calkins T, Tybout AM, Calkins T (2005). The challenge of branding. Kellogg on Branding: The Marketing Faculty of The Kellogg School of Management.

[6] Centers for Disease Control and Prevention (2014). Best Practices for Comprehensive Tobacco Control Programs—2014.

[7] Ferner M (2014). Huffington Post.

[8] Jager G, Block RI, Luijten M, Ramsey NF (2010). Cannabis use and memory brain function in adolescent boys: A cross-sectional multicenter functional magnetic resonance imaging study. J Am Acad Child Adolesc Psychiatry.

[9] Medina KL, Schweinsburg AD, Cohen-Zion M, Nagel BJ, Tapert SF (2007). Effects of alcohol and combined marijuana and alcohol use during adolescence on hippocampal volume and asymmetry. Neurotoxicol Teratol.

[10] Durkin A (2014). Legalization of marijuana for non-medical use: Health, policy, socioeconomic, and nursing implications. J Psychosoc Nurs Ment Health Serv.

[11] Pedersen ER, Miles JN, Ewing BA, Shih RA, Tucker JS, D'Amico EJ (2013). A longitudinal examination of alcohol, marijuana, and cigarette perceived norms among middle school adolescents. Drug Alcohol Depend.

[12] Walker DD, Neighbors C, Rodriguez LM, Stephens RS, Roffman RA (2011). Social norms and self-efficacy among heavy-using adolescent marijuana smokers. Psychol Addict Behav.

[13] Friese B, Grube JW (2013). Legalization of medical marijuana and marijuana use among youths. Drugs (Abingdon Engl).

[14] Cerdá M, Wall M, Feng T, Keyes KM, Sarvet A, Schulenberg J, O'Malley PM, Pacula RL, Galea S, Hasin DS (2017). Association of state recreational marijuana laws with adolescent marijuana use. JAMA Pediatr.

[15] Living the Example.

[16] Free C, Phillips G, Galli L, Watson L, Felix L, Edwards P, Patel V, Haines A (2013). The effectiveness of mobile-health technology-based health behaviour change or disease management interventions for health care consumers: A systematic review. PLoS Med.

[17] Tomlinson M, Rotheram-Borus MJ, Swartz L, Tsai AC (2013). Scaling up mHealth: Where is the evidence?. PLoS Med.

[18] Evans WD, Holtz K, White T, Snider J (2014). Effects of the above the influence brand on adolescent drug use prevention normative beliefs. J Health Commun.

[19] Andrade E, Evans W, Edberg M, Cleary SD, Villalba R, Batista IC (2015). Victor and Erika webnovela: An innovative generation @ audience engagement strategy for prevention. J Health Commun.

[20] (2015). Substance Abuse and Mental Health Services Administration (SAMHSA).

[21] Substance Abuse and Mental Health Services Administration (SAMHSA).

[22] Snyder L, Hamilton M, Mitchell E, Kiwanuka-Tondo J, Fleming-Milici F, Proctor D (2004). A meta-analysis of the effect of mediated health communication campaigns on behavior change in the United States. J Health Commun.

[23] Ennett ST, Tobler NS, Ringwalt CL, Flewelling RL (1994). How effective is drug abuse resistance education? A meta-analysis of Project DARE outcome evaluations. Am J Public Health.

[24] Gottfredson D, Wilson D (2003). Characteristics of effective school-based substance abuse prevention. Prev Sci.

[25] Skara S, Sussman S (2003). A review of 25 long-term adolescent tobacco and other drug use prevention program evaluations. Prev Med.

[26] Spoth R, Greenberg M, Turrisi R (2008). Preventive interventions addressing underage drinking: State of the evidence and steps toward public health impact. Pediatrics.

[27] Hecht M, Marsiglia F, Elek E, Wagstaff D, Kulis S, Dustman P, Miller-Day M (2003). Culturally grounded substance use prevention: An evaluation of the keepin' it REAL curriculum. Prev Sci.

[28] Slater MD, Kelly KJ, Edwards RW, Thurman PJ, Plested BA, Keefe TJ, Lawrence FR, Henry KL (2006). Combining in-school and community-based media efforts: Reducing marijuana and alcohol uptake among younger adolescents. Health Educ Res.

[29] Perry CL, Williams CL, Veblen-Mortenson S, Toomey TL, Komro KA, Anstine PS, McGovern PG, Finnegan JR, Forster JL, Wagenaar AC, Wolfson M (1996). Project Northland: Outcomes of a community-wide alcohol use prevention program during early adolescence. Am J Public Health.

[30] Colby M, Hecht M, Miller-Day M, Krieger J, Syvertsen AK, Graham J, Pettigrew J (2013). Adapting school-based substance use prevention curriculum through cultural grounding: A review and exemplar of adaptation processes for rural schools. Am J Community Psychol.

[31] Pettigrew J, Graham JW, Miller-Day M, Hecht ML, Krieger JL, Shin YJ (2015). Adherence and delivery: Implementation quality and program outcomes for the seventh-grade keepin' it REAL program. Prev Sci.

[32] Chou W, Hunt Y, Beckjord E, Moser R, Hesse B (2009). Social media use in the United States: Implications for health communication. J Med Internet Res.

[33] Pidgeon N, Henwood K, Maguire B, Bennet P, Calman K (1999). Public health communication and the social amplification of risks: Present knowledge and future prospects. Risk Communication and Public Health.

[34] Bandura A (2004). Health promotion by social cognitive means. Health Educ Behav.

[35] Fishbein M, Ajzen I (2010). Predicting and Changing Behavior: The Reasoned Action Approach.

[36] Evans WD, Evans WD (2013). Branding social and health behavior: An education and research agenda. Psychology of Branding.

[37] Evans WD, Rath J, Vallone D, Cantrell J, Pitzer L, Hair E (2017). Effects of the truth FinishIt brand on adolescent and young adult tobacco use outcomes (in press). Am J Prev Med.

[38] Evans W, Rath J, Pitzer L, Hair E, Snider J, Cantrell J, Vallone D (2016). Design and feasibility testing of the truth FinishIt tobacco countermarketing brand equity scale. J Health Commun.

[39] Substance Abuse and Mental Health Services Administration (SAMHSA).

[40] Johnston LD, O'Malley PM, Miech RA, Bachman JG, Schulenberg JE (2015). Monitoring the Future National Survey Results on Drug Use, 1975-2014: Overview—Key Findings on Adolescent Drug Use.

[41] D'Amico EJ, Miles JN, Tucker JS (2015). Gateway to curiosity: Medical marijuana ads and intention and use during middle school. Psychol Addict Behav.

[42] DuRant RH, Smith JA, Kreiter SR, Krowchuk DP (1999). The relationship between early age of onset of initial substance use and engaging in multiple health risk behaviors among young adolescents. Arch Pediatr Adolesc Med.

[43] Merrill RM (2015). Use of marijuana and changing risk perceptions. Am J Health Behav.

